# Narcolepsy-like presentations in intellectual disability with comorbid epilepsy and obstructive sleep apnea: a case-based diagnostic analysis and literature-informed discussion

**DOI:** 10.3389/fpsyt.2026.1919011

**Published:** 2026-09-04

**Authors:** Shuning Hong, Lili Yang, Lishan Ren, Haisi Chen, Yuping Zheng, Wenjuan Liu

**Affiliations:** 1 Affiliated Mental Health Center & Hangzhou Seventh People’s Hospital, Zhejiang University School of Medicine, Hangzhou, China; 2 The Fifth People’s Hospital of Lin’an District, Hangzhou, China

**Keywords:** diagnostic overshadowing, epilepsy, excessive daytime sleepiness, intellectual disability, narcolepsy, obstructive sleep apnea

## Abstract

Excessive daytime sleepiness (EDS), nocturnal impaired-awareness episodes, and abnormal sleep-related behaviors have complex and overlapping etiologies. Narcolepsy, obstructive sleep apnea (OSA), and epileptic nocturnal events may present with similar clinical manifestations. In patients with intellectual disability (ID), restricted self-reporting, incomplete caregiver observations, and diagnostic overshadowing may lead clinicians to misattribute underlying neurological or sleep disorders to preexisting cognitive impairment or psychiatric and behavioral problems, resulting in delayed diagnosis or misdiagnosis. We present an illustrative case of a 34-year-old woman with lifelong cognitive and adaptive impairments who was referred for long-standing snoring, EDS, and two nocturnal impaired-awareness episodes. An outside emergency department had previously considered “sleep apnea?” and “narcolepsy?” as possible diagnoses. After detailed collateral history-taking, overnight polysomnography (PSG), the Multiple Sleep Latency Test (MSLT), and two 24-hour ambulatory electroencephalography (EEG) recordings, she was diagnosed with epilepsy with focal to bilateral tonic-clonic seizures, moderate OSA, and mild ID. At the 6-month follow-up after concurrent initiation of levetiracetam (LEV) and continuous positive airway pressure (CPAP) therapy, no further nocturnal impaired-awareness episodes, limb jerking, or cyanosis of the lips were reported; her EDS also improved, with the Epworth Sleepiness Scale (ESS) score decreasing from 18/24 at baseline to 6/24. Rather than reporting a rare comorbidity alone, this case-based diagnostic analysis uses the index case to clarify the differential diagnostic approach to EDS and nocturnal events in patients with ID, highlights the key distinctions among narcolepsy, OSA-related sleepiness, and epileptic nocturnal events, and emphasizes the need for integrated management when OSA and epilepsy coexist.

## 1 Introduction

Excessive daytime sleepiness (EDS) is a common but nonspecific manifestation in sleep medicine and neuropsychiatric practice. Beyond narcolepsy, obstructive sleep apnea (OSA), epileptic nocturnal events, and other causes of sleep disruption can all lead to daytime sleepiness or reduced alertness. When nocturnal abnormal breathing, transient impaired awareness, limb jerking, or postictal confusion co-occur with EDS, diagnosing narcolepsy based solely on “sleepiness” may obscure other identifiable and treatable causes.

The diagnostic process is even more challenging in patients with intellectual disability (ID). These patients often have difficulty accurately describing preictal experiences, nocturnal symptoms, and postictal states, making clinical assessment highly dependent on eyewitness accounts from family members or caregivers. At the same time, daytime sleepiness, slowed responses, behavioral changes, or functional decline may be interpreted as part of the preexisting cognitive impairment, leading to diagnostic overshadowing. Therefore, when evaluating these patients, clinicians should not focus only on long-standing cognitive and adaptive impairments, but should also actively determine whether the presenting symptoms represent a change from the patient’s previous baseline.

We present a patient with mild intellectual disability whose long-standing EDS, abnormal breathing during sleep, and two nocturnal impaired-awareness episodes initially raised clinical suspicion for narcolepsy. Further evaluation established a diagnosis of epilepsy comorbid with OSA. Using this case as a clinical anchor, this article analyzes the diagnostic pitfalls of narcolepsy-like presentations in patients with ID, highlights the differential diagnosis among narcolepsy, OSA-related sleepiness, and epileptic nocturnal events, and summarizes a practical evaluation pathway for similar patients.

## 2 Case presentation

### 2.1 Patient information

The patient was a 34-year-old woman with a body mass index (BMI) of 28.40 kg/m² who was admitted for “long-standing daytime sleepiness and two nocturnal impaired-awareness episodes.” In recent years, she had developed prominent nocturnal snoring, which her family described as loud and irregular in rhythm. During the daytime, she frequently yawned and became easily drowsy. After an outside emergency department considered “sleep apnea” and “narcolepsy?” as possible diagnoses, the patient and her family came to our hospital for further evaluation.

### 2.2 Nocturnal events and collateral history

Further inquiry with the family revealed that the patient had experienced two stereotyped nocturnal events.

The first event occurred during nighttime sleep in July 2022. Her family noticed abnormal snoring, unresponsiveness, and a small amount of blood-tinged oral secretions. Because the episode resolved quickly, no systematic evaluation or treatment was pursued at that time.

The second event occurred during nocturnal sleep in November 2025. During sleep, the patient first developed abnormal snoring and a pause in breathing, followed by cyanosis of the lips and face and unresponsiveness. Her family observed unilateral limb jerking at onset, which subsequently progressed to bilateral limb jerking, generalized muscle rigidity, a small amount of foamy oral secretions, tongue rigidity, and upward deviation of the eyes. The event lasted approximately 2–3 minutes. After the jerking stopped, she sat up purposelessly, showed groping hand movements, and attempted to walk. After she returned to baseline awareness, she reported fatigue, dizziness, chest tightness, nausea, and vomiting, and had no recollection of the event.

### 2.3 Developmental and cognitive assessment

The patient had several perinatal risk factors. According to her family, the delivery was complicated, and she developed a persistent high fever during the first 3 days of life. Because of limited medical resources at that time, she did not receive timely systematic evaluation or treatment. Since childhood, she had delayed language and learning development. She began speaking at approximately 3 years of age and dropped out of school after the third grade of primary school. In adulthood, she had never held formal employment and currently helped with simple tasks at her family’s breakfast shop. She still required assistance from family members with daily activities such as laundry and cooking.

After admission, the Wechsler Adult Intelligence Scale showed a full-scale intelligence quotient of 56, and adaptive behavior assessment indicated overall adaptive functioning below the expected level for her age. Taken together with her developmental history and current functional status, these findings supported a long-standing neurodevelopmental impairment consistent with mild intellectual disability. This neurodevelopmental background may be relevant not only to her intellectual disability but also to her vulnerability to epileptic seizures.

### 2.4 PSG and MSLT findings

Overnight polysomnography (PSG) was performed using a standard clinical sleep-monitoring protocol. The report provided sleep staging, arousal scoring, respiratory event analysis, oxygen saturation, heart rate, body position, limb movements, and snoring-related parameters. EEG signals were used for sleep staging and arousal scoring, but the recording did not include an extended epilepsy EEG montage. Therefore, nocturnal EEG activity during PSG was not used for formal assessment of epileptiform abnormalities.

The PSG showed a sleep latency of 8.5 minutes, a total sleep time of 502 minutes, and a sleep efficiency of 91.4%. The patient spent 72.1% of sleep time in the supine position. The apnea-hypopnea index (AHI) was 19.7 events/hour, including 68 obstructive apneas, 29 hypopneas, and 68 central apneas. The lowest oxygen saturation was 83%, and the arousal index was 28.2 events/hour, mainly related to respiratory events. The snoring index was markedly elevated.

Taken together, the presence of prominent snoring, obstructive apneas, hypopneas, nocturnal oxygen desaturation, a high proportion of supine sleep, and respiratory-related arousals supported the diagnosis of moderate OSA. At the same time, PSG also recorded a relatively high number of central apneas. This finding suggested that sleep instability, altered respiratory control, or epilepsy-related breathing abnormalities might also have contributed to the respiratory pattern. Because combined video-EEG/PSG monitoring was not performed in this case, the temporal relationship between central apneas and epileptiform discharges or nocturnal seizures could not be determined.

The multiple sleep latency test (MSLT) showed a mean sleep latency of 9.3 minutes across four naps, with no sleep-onset rapid eye movement (REM) periods (SOREMPs). No SOREMPs were recorded during the preceding overnight PSG. Because the MSLT was performed in the presence of untreated moderate OSA, the results were interpreted cautiously, as untreated sleep-disordered breathing may influence MSLT interpretation. The patient also lacked typical REM-related symptoms, including cataplexy, sleep paralysis, and hypnagogic or hypnopompic hallucinations. Therefore, narcolepsy was considered unlikely based on the overall clinical assessment, including the clinical history, absence of typical REM-related symptoms, PSG, MSLT, and subsequent EEG findings, rather than on the MSLT results alone. Her daytime sleepiness was considered more likely to be related to OSA-associated sleep fragmentation and nocturnal hypoxemia, potentially compounded by sleep disruption associated with epilepsy.

### 2.5 EEG findings and diagnosis

**Figure 1:**
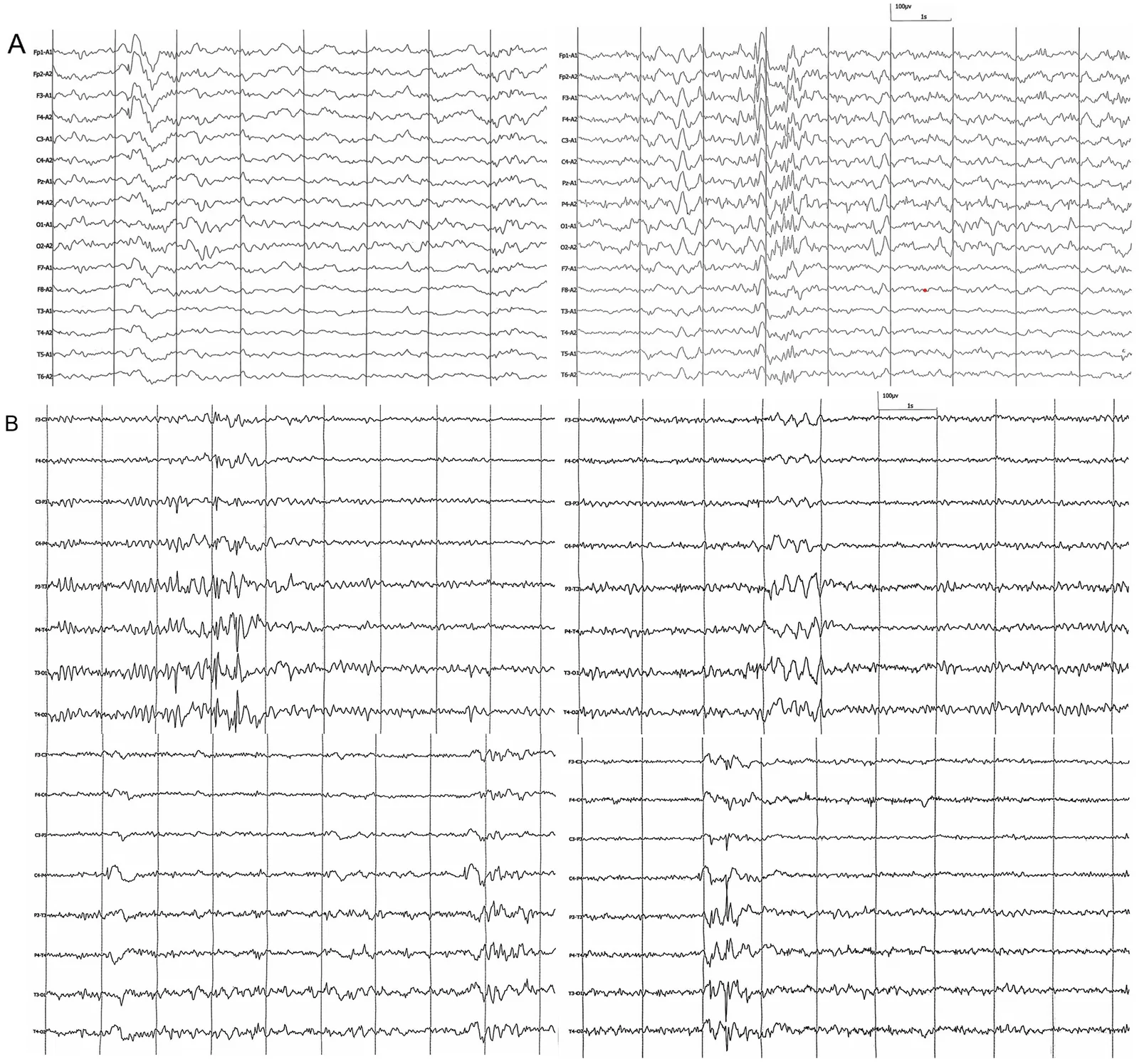
24-hour ambulatory EEG findings. **(A)** Initial recording showing sleep-activated epileptiform discharges. **(B)** Repeat recording showing persistent epileptiform abnormalities.

The patient underwent two 24-hour ambulatory electroencephalography (EEG) recordings. The first recording showed epileptiform discharges, including sharp-and-slow waves and spike-and-slow waves, during both wakefulness and sleep, with a marked increase during sleep. These discharges predominantly involved the frontal region, right frontal region, and part of the parietal region (**Figure 1A**). Repeat EEG still showed intermittent spike-and-slow waves most prominently over the right frontocentral region, as well as generalized high-amplitude spike-and-slow waves and slow-wave activity (**Figure 1B**). These findings supported the diagnosis of epilepsy.

Brain MRI with diffusion-weighted imaging was performed to evaluate possible structural etiologies of the seizures, and no definite structural epileptogenic lesion was identified. The diagnosis fulfilled the ILAE practical clinical definition of epilepsy because the patient had recurrent apparently unprovoked nocturnal seizures occurring more than 24 hours apart, with sleep-activated epileptiform discharges further supporting the diagnosis. Based on the clinical history, PSG, MSLT, ambulatory EEG, and MRI findings, the final diagnoses were: (1) epilepsy with focal to bilateral tonic-clonic seizures; (2) moderate obstructive sleep apnea; and (3) mild intellectual disability.

### 2.6 Treatment and follow-up

**Figure 2:**
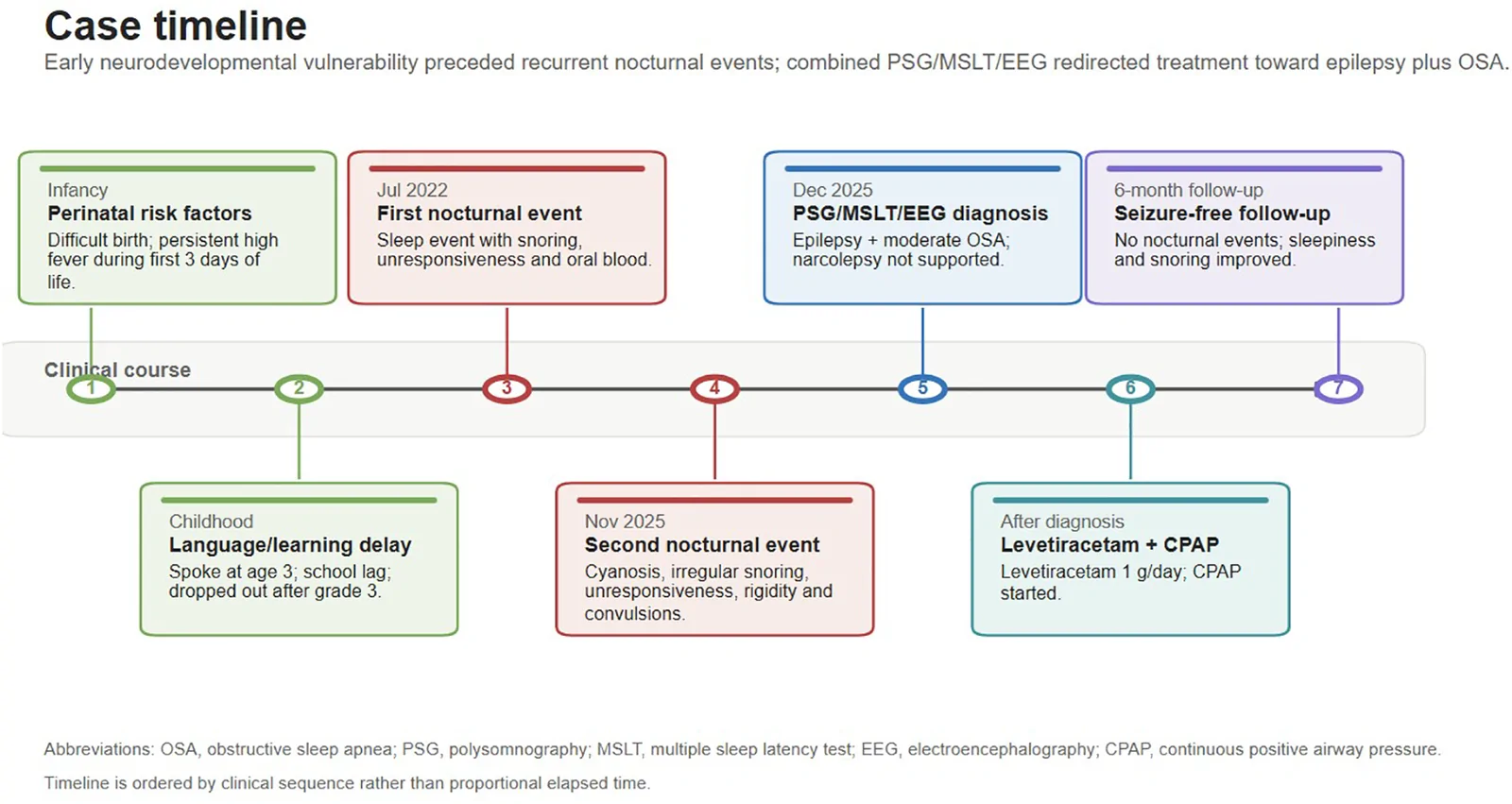
Timeline of the patient’s clinical course.

After the diagnosis was established in routine clinical care, the patient was started on levetiracetam (LEV) at 500 mg twice daily and continuous positive airway pressure (CPAP) therapy. She was also advised to pursue weight management and improve sleep hygiene. Follow-up information over the subsequent 6 months was retrospectively obtained from clinical records and caregiver reports. No further nocturnal episodes of impaired awareness, limb jerking, or cyanosis of the lips were reported. Her family observed a marked reduction in snoring, more stable breathing, and substantial improvement in EDS and fatigue. The baseline Epworth Sleepiness Scale (ESS) score was 18/24, indicating marked daytime sleepiness, and decreased to 6/24 at the 6-month follow-up. Her participation in daily family activities also improved compared with baseline. However, apart from ESS reassessment, repeat PSG or follow-up EEG was not performed; therefore, changes in sleep-disordered breathing and epileptiform activity could not be objectively reassessed. A detailed timeline of the case is presented in **Figure 2**.

## 3 Discussion

### 3.1 Diagnostic pitfalls

Although this case involved mild intellectual disability, epilepsy, and OSA, its main clinical value lies less in the rarity of the comorbidity than in the diagnostic pathway that may lead clinicians away from treatable causes. When EDS, abnormal nocturnal breathing, and transient impaired awareness are the main presenting features, the diagnostic focus can easily shift among narcolepsy, OSA, and epileptic nocturnal events. According to the International Classification of Sleep Disorders, Third Edition, Text Revision (ICSD-3-TR), the diagnosis of narcolepsy should not be based solely on daytime sleepiness, but should integrate typical clinical features with objective sleep test findings, particularly whether the mean sleep latency is shortened on the MSLT and whether SOREMPs are present (1). The American Academy of Sleep Medicine (AASM) recommendations for MSLT protocols also emphasize that interpretation of MSLT results should take into account the adequacy of nocturnal sleep, medication effects, and whether OSA has been treated (2, 3).

EDS may occur in narcolepsy, OSA, and epileptic nocturnal events, but the underlying pathophysiological mechanisms and diagnostic criteria differ among these conditions. Narcolepsy is characterized by irresistible sleep attacks, REM-related symptoms, and supportive MSLT findings (4). OSA-related sleepiness is mainly associated with recurrent upper-airway collapse, intermittent hypoxemia, respiratory-related arousals, and sleep fragmentation during sleep; its diagnosis relies on comprehensive PSG assessment of indices such as elevated AHI, oxygen desaturation, snoring, arousals, or respiratory effort-related arousals (5). Epileptic nocturnal events, by contrast, rely more heavily on the stereotyped and brief nature of events, postictal features, and EEG evidence (6). The key differentiating features among the three conditions are summarized in **Table 1**.

**Table 1:** Key differentiating features among narcolepsy, OSA-related sleepiness, and epileptic nocturnal events.

Differentiating feature	Narcolepsy	OSA-related sleepiness	Epileptic nocturnal events
Core presentation	Central hypersomnolence with recurrent, irresistible sleep attacks	Sleepiness related to recurrent obstructive respiratory events	Stereotyped nocturnal or sleep–wake transition seizures
Key nocturnal/event features	Sleep fragmentation; possible sleep paralysis or hypnagogic/hypnopompic hallucinations	Snoring, witnessed apneas, choking arousals, nocturnal hypoxemia	Stereotyped motor events, automatisms, cyanosis, tongue biting, and postictal confusion
Objective evidence	MSLT (2): mean sleep latency ≤8 min and ≥2 SOREMPs	PSG (11): elevated AHI, hypoxemia, and arousals	EEG/video-EEG (12): sleep-activated epileptiform discharges
Treatment focus	Sleep hygiene, scheduled naps, and wake-promoting or anticataplectic medications	CPAP, weight management, positional or upper-airway therapy	Antiseizure medication; treat comorbid OSA when present

The initial consideration of “narcolepsy?” was mainly driven by the patient’s long-standing EDS. However, the MSLT showed a mean sleep latency of 9.3 minutes without SOREMPs, and she lacked typical REM-related symptoms, including cataplexy, sleep paralysis, and hypnagogic or hypnopompic hallucinations. Thus, narcolepsy was unlikely to be the most appropriate explanation for her symptoms (4, 7). In contrast, PSG showed an AHI of 19.7 events/hour, a lowest oxygen saturation of 83%, and prominent snoring, supporting moderate OSA. Two 24-hour ambulatory EEG recordings showed epileptiform abnormalities during both wakefulness and sleep, with marked sleep activation. Together with cyanosis, transient unresponsiveness, progression from unilateral limb jerking to bilateral tonic-clonic movements, blood-tinged oral secretions, postictal automatisms, and amnesia, these findings supported epilepsy with focal to bilateral tonic-clonic seizures occurring during sleep (8).

Sleep-related hypermotor epilepsy (SHE) was considered because of the sleep-related onset, sleep-activated epileptiform abnormalities, and frontal EEG involvement. However, typical SHE was less likely because the events were rare, not clustered, and were dominated by tonic-clonic evolution and postictal features rather than brief recurrent hypermotor behaviors or dystonic-tonic posturing. Nevertheless, SHE could not be completely excluded without combined video-EEG/PSG capture of a habitual event.

Other sleep-related motor disorders were also considered. Non-rapid eye movement parasomnias may present with incomplete arousal, confusion, and amnesia, but they would not readily explain cyanosis, blood-tinged oral secretions, progression to bilateral tonic-clonic movements, postictal automatisms, or sleep-activated epileptiform discharges. REM sleep behavior disorder was considered less likely because there was no history of dream-enactment behavior and the PSG report did not indicate REM sleep without atonia. Sleep-related movement disorders were also less likely because they typically cause repetitive movements or sleep fragmentation rather than impaired awareness, postictal features, or epileptiform EEG abnormalities.

At least three diagnostic pitfalls were present in this case. First, prominent EDS made narcolepsy an early consideration in the differential diagnosis, but the MSLT findings and absence of REM-related symptoms did not support this diagnosis as the primary explanation. Second, because the nocturnal events occurred during sleep and were accompanied by abnormal snoring, apnea, and hypoxemia, they could initially be misinterpreted as isolated OSA-related arousals or choking episodes. However, the tonic-clonic manifestations, postictal automatisms, and amnesia could not be adequately explained by a purely respiratory event and were more suggestive of epileptic seizures (9). Third, the patient had intellectual disability since childhood; newly emerging sleepiness, slowed responses, functional decline, or abnormal nocturnal behaviors could therefore be misattributed to preexisting cognitive impairment, contributing to diagnostic overshadowing (10). Thus, when EDS coexists with nocturnal impaired-awareness episodes, PSG, MSLT, and EEG findings may be best interpreted within a unified diagnostic framework, rather than focusing only on whether the condition is narcolepsy.

### 3.2 Diagnostic overshadowing in patients with intellectual disability

Diagnostic overshadowing refers to the tendency of clinicians to attribute newly emerging physical, neurological, or psychiatric symptoms directly to a patient’s preexisting disability, psychiatric condition, or cognitive impairment, thereby overlooking comorbid conditions that could otherwise be identified and treated (13, 14). In recent years, sleep problems in adults with intellectual disability have received increasing attention; however, related sleep disorders remain underrecognized, with inconsistent diagnostic pathways and limited evidence for intervention (10, 15). From the perspective of sleep-disordered breathing, substantial diagnostic and treatment gaps also persist for OSA and related sleep-related breathing problems in both children and adults with intellectual disability (16). In addition, studies by Dell’Armo and Tassé on diagnostic overshadowing in intellectual disability suggest that intellectual disability itself may interfere with clinicians’ interpretation of comorbid symptoms, leading psychological, neurological, or somatic disorders to be underestimated or misattributed (13, 14). Detailed history from relatives or caregivers is therefore crucial and may help correct previous misdiagnosis (17).

The present case illustrates this issue. The patient had language, learning, and adaptive impairments since childhood and continued to require assistance from family members with some activities of daily living in adulthood. As a result, she had difficulty accurately describing preictal experiences, a sense of choking during the event, postictal discomfort, or changes in the severity of daytime sleepiness. Clinical assessment therefore depended heavily on caregiver descriptions of nocturnal events and recent functional changes. For similar patients, evaluation should not rely solely on static descriptions such as “long-standing cognitive impairment” or “poor adaptive functioning.” Instead, clinicians should actively determine whether current symptoms represent a deviation from the previous baseline. New or worsening daytime sleepiness, sleep-related breathing abnormalities, or nocturnal paroxysmal events should prompt evaluation for neurological or sleep-related disorders, rather than being attributed simply to intellectual disability itself.

### 3.3 OSA–epilepsy interaction

OSA and epilepsy are not merely coexisting conditions; rather, they may form a mutually reinforcing pathophysiological loop. Studies have shown that OSA is relatively common among patients with epilepsy and may affect seizure control through sleep fragmentation, intermittent hypoxemia, and recurrent arousals, whereas CPAP therapy may improve seizure control in some patients (18, 19). Pavlova et al. also emphasized that OSA may influence epilepsy treatment outcomes and that screening for sleep-disordered breathing should be incorporated into the evaluation of patients with drug-resistant epilepsy, predominantly nocturnal seizures, or prominent snoring and daytime sleepiness (19).

Mechanistically, OSA may lower the seizure threshold through several pathways. Recurrent apneas and hypopneas can lead to intermittent hypoxemia, hypercapnia, and sleep fragmentation. Frequent arousals may further disrupt sleep continuity, amplify the effects of sleep deprivation, and promote seizures through sympathetic activation, oxidative stress, neuroinflammation, and increased cortical excitability (20, 21). Conversely, nocturnal seizures can disrupt sleep continuity. Postictal impaired awareness, reduced arousal responses, and decreased upper-airway muscle tone may also make preexisting respiratory events more difficult to terminate promptly (22, 23). Some patients may also develop peri-ictal or postictal abnormalities in respiratory control, further aggravating hypoxemia and sleep-related breathing events (24, 25). Therefore, OSA and epilepsy may form a vicious cycle of “hypoxemia–sleep fragmentation–increased cortical excitability–nocturnal seizures–further sleep and respiratory instability.”

The findings in this patient were consistent with the mechanisms described above. PSG showed moderate OSA, with a lowest oxygen saturation of 83% and an elevated arousal index, whereas ambulatory EEG showed increased epileptiform discharges during sleep. These findings suggest that OSA-related hypoxemia and sleep fragmentation may have lowered the patient’s seizure threshold, while nocturnal epileptic seizures may have further disrupted sleep architecture and respiratory stability.

### 3.4 Central apnea and respiratory risk

In this case, PSG recorded obstructive respiratory events as well as a number of central apneas. This finding should be interpreted cautiously in the context of coexisting epilepsy and sleep-disordered breathing. Central apneas observed on PSG do not necessarily indicate primary central sleep apnea syndrome. In patients with epilepsy, ictal or postictal apnea may be related to the effects of epileptic discharges on respiratory control networks (26).

In recent years, peri-ictal respiratory abnormalities have become an important focus in research on the mechanisms of sudden unexpected death in epilepsy (SUDEP). Epileptic seizures may be accompanied by ictal or postictal central apnea, and these phenomena may involve the limbic system, paralimbic structures, and brainstem respiratory control networks. At the anatomical pathway level, Lacuey et al. induced central apnea by stimulating limbic structures, including the amygdala, thereby supporting the existence of a medial temporal limbic/paralimbic respiratory control network in the human brain (27). When epileptic discharges originate from or propagate to these regions, they may interfere with brainstem-mediated autonomic respiratory drive. At the clinical pathophysiological level, previous studies have suggested that severe hypoxemia caused by postictal central apnea, together with generalized EEG suppression, may represent a terminal pathophysiological process leading to SUDEP and may reflect profound postictal suppression of brainstem function (28, 29). In addition, a recent breath-hold functional magnetic resonance imaging study by Ciumas et al. showed that, under respiratory challenge, patients with epilepsy exhibited reduced activation of brainstem respiratory-related nuclei and altered cortico-brainstem functional connectivity, further suggesting impairment of respiratory control networks in epilepsy (26).

Although SUDEP did not occur in this case and long-term risk cannot be inferred from a single PSG study alone, the patient experienced cyanosis, abnormal breathing, and impaired awareness during nocturnal seizures, together with OSA-related hypoxemia. These findings suggest that respiratory monitoring should receive greater attention in similar patients. It should be emphasized that, based on the PSG findings, the overall sleep-disordered breathing profile in this case was more consistent with moderate OSA, although the burden of central apneas should not be overlooked. The presence of prominent snoring, obstructive apneas, hypopneas, nocturnal oxygen desaturation, and respiratory-related arousals supported recurrent upper-airway obstruction. However, the central apneas recorded on PSG may have reflected sleep instability, altered ventilatory control, or epilepsy-related respiratory dysregulation. Because combined video-EEG/PSG monitoring was not performed, the temporal relationships among central apneas, oxygen desaturation, and epileptiform activity could not be determined. In similar cases, combined video-EEG/PSG monitoring may help clarify these relationships and inform subsequent treatment and risk management.

### 3.5 Medication and integrated management

When epilepsy coexists with OSA, antiseizure medication should be selected with attention to sedation, respiratory function, cognition, and daytime alertness. In the present patient, these considerations were particularly relevant because she had prominent EDS, moderate OSA, and intellectual disability. Levetiracetam (LEV) was selected because previous studies suggest that it has relatively limited effects on sleep architecture and cognition, a low sedative burden, and few pharmacokinetic interactions (30–34). Nevertheless, neuropsychiatric adverse effects, including irritability, mood changes, and depression, should still be monitored, particularly in patients with cognitive or behavioral vulnerability (35, 36).

OSA management should also be integrated into epilepsy care. Weight reduction, sleep hygiene, and lifestyle modification remain fundamental, while continuous positive airway pressure (CPAP) is an important therapy for moderate to severe OSA. In patients with comorbid epilepsy and OSA, OSA treatment may improve snoring, nocturnal hypoxemia, and EDS, and may also contribute to better seizure control. Earlier reports and retrospective studies suggest that CPAP may reduce seizure frequency in some patients by decreasing nocturnal hypoxemia, improving sleep continuity, reducing arousals, and alleviating sleep deprivation (18, 37). Jo et al. reported that 74.9% of patients with epilepsy who underwent overnight PSG were diagnosed with OSA, and approximately 66.0% of these cases were moderate to severe (18). However, because snoring, witnessed apnea, or daytime sleepiness may be atypical or underreported, OSA is often underestimated or missed. Sleep history-taking, OSA screening, and timely treatment should therefore be incorporated into comprehensive epilepsy management (19, 38).

After treatment with LEV combined with CPAP therapy, the patient had no further nocturnal impaired-awareness episodes, limb jerking, or cyanosis of the lips. Her EDS and fatigue improved substantially, and her family observed less nocturnal snoring. Because antiseizure treatment and OSA intervention were initiated simultaneously, this single case cannot distinguish the individual contributions of LEV and CPAP or establish their independent therapeutic effects. Nevertheless, the clinical course suggests that concurrent recognition and management of epilepsy and OSA better align with the patient’s pathophysiology and therapeutic needs. Focusing only on epilepsy may leave nocturnal hypoxemia and sleep fragmentation insufficiently addressed, whereas attributing nocturnal events solely to OSA may delay epilepsy diagnosis and secondary prevention.

### 3.6 Diagnostic pathway

**Figure 3:**
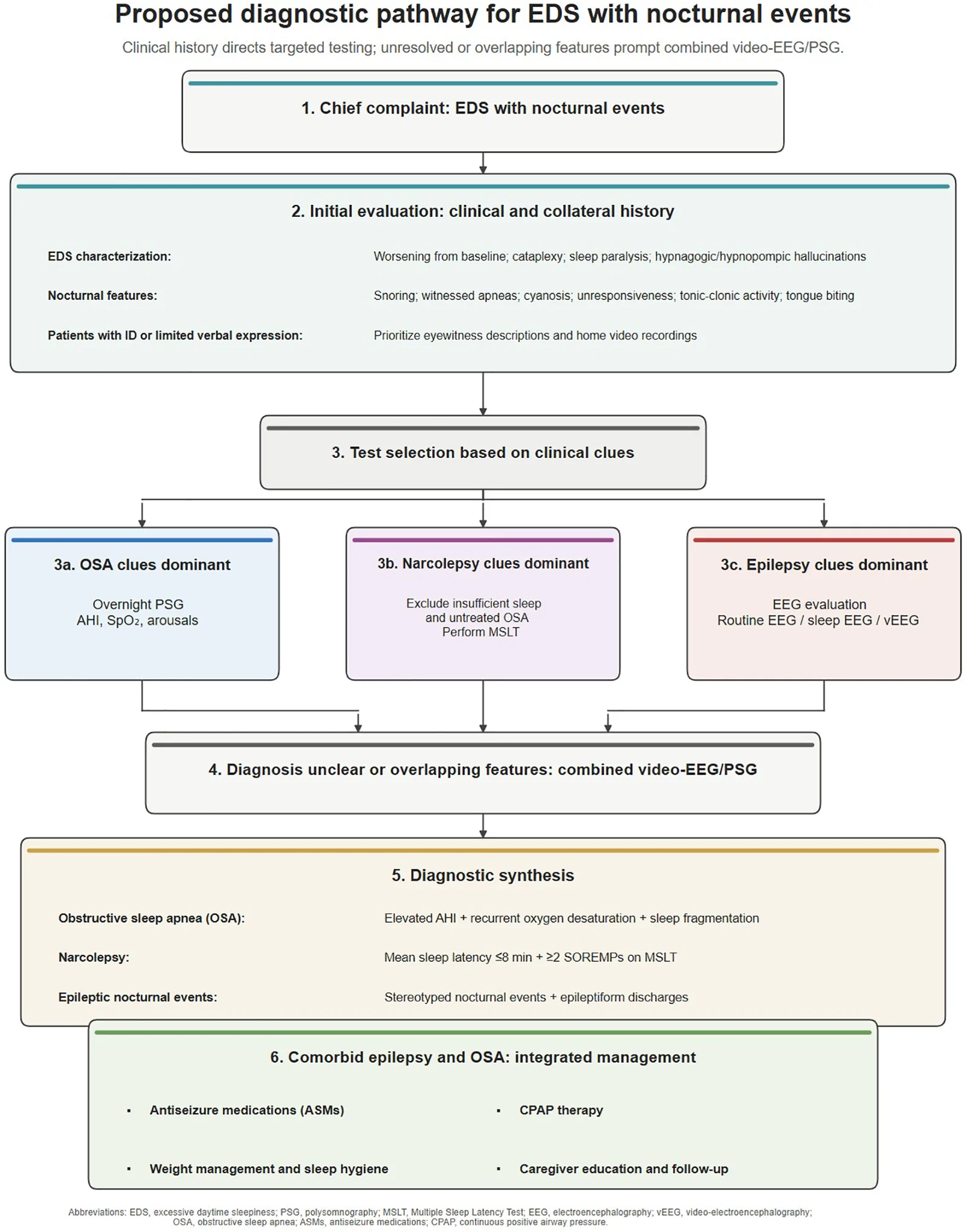
Proposed diagnostic pathway for EDS with nocturnal events.

For patients who present with both EDS and nocturnal events, the diagnostic approach should not begin with the single question of whether the condition is narcolepsy. Instead, clinicians should first clarify the nature of the patient’s daytime sleepiness and then assess whether the nocturnal events suggest OSA, epileptic nocturnal events, or overlapping conditions. The patient’s reported “sleepiness” may represent irresistible sleep attacks, but it may also reflect fatigue, reduced attention, or slowed responses resulting from repeated disruption of nocturnal sleep. For patients with intellectual disability or limited verbal expression, collateral history is particularly important. Clinicians should determine whether recent symptoms deviate from the previous baseline and document breathing changes, cyanosis, stereotyped limb movements, event duration, postictal features, and amnesia during nocturnal events. Family-provided videos may also be useful. A proposed stepwise diagnostic pathway for patients presenting with EDS and nocturnal events is shown in **Figure 3**.

Diagnostic tests should be selected in a stepwise manner according to clinical clues. In patients with snoring, witnessed apnea, obesity, or nocturnal hypoxemia, overnight PSG may be useful for evaluating sleep-disordered breathing. If narcolepsy remains clinically suspected, MSLT may be considered after untreated OSA, insufficient sleep, and medication effects have been excluded. For patients with nocturnal impaired-awareness episodes, stereotyped motor activity, postictal amnesia, or other red flags for epilepsy, EEG evaluation may provide important diagnostic information; when necessary, sleep EEG, ambulatory EEG, or video-EEG monitoring may be considered. When the nature of nocturnal events remains unclear, combined video-EEG/PSG monitoring can simultaneously record EEG activity, behavioral manifestations, respiratory events, and oxygen saturation changes. The final diagnosis should be based on the integration of clinical history, caregiver observations, and objective test findings. Daytime sleepiness is not synonymous with narcolepsy, and nocturnal snoring does not exclude epilepsy. For patients with comorbid OSA and epilepsy, concurrent management is needed to reduce their reciprocal effects.

### 3.7 Limitations

This study has several limitations. First, as a retrospective single-case analysis, it cannot establish causal relationships among OSA, epilepsy, treatment, and clinical improvement. Because LEV and CPAP therapy were initiated concurrently, the absence of further nocturnal events and improvement in daytime symptoms should be interpreted cautiously rather than as evidence that either intervention independently improved seizure control. Second, the initial PSG was designed primarily to assess sleep architecture and sleep-disordered breathing and did not include an extended EEG montage for comprehensive evaluation of epileptiform activity. Moreover, no habitual nocturnal event was captured; therefore, the temporal relationship among respiratory events, epileptiform activity, and nocturnal semiology could not be determined. Third, follow-up relied mainly on clinical records and caregiver reports because the patient had moved outside the province and was unable to return for in-person reassessment. CPAP device-download data, residual AHI, repeat PSG, follow-up EEG, and combined video-EEG/PSG monitoring were therefore not available, limiting objective assessment of sleep-disordered breathing, CPAP efficacy, and epileptiform activity.

## 4 Conclusion

This case highlights that EDS and nocturnal events in patients with intellectual disability should not be attributed automatically to narcolepsy or baseline cognitive impairment. When loud snoring, nocturnal hypoxemia, cyanosis, impaired awareness, tonic-clonic movements, blood-tinged oral secretions, postictal automatisms, or amnesia are present, both OSA and epileptic nocturnal events should be considered. Our case suggests that integrated interpretation of clinical history, PSG, MSLT, and EEG may be valuable in selected patients with intellectual disability presenting with EDS and nocturnal events. The clinical improvement observed after concurrent treatment further supports the importance of recognizing and managing comorbid epilepsy and OSA together, although causal inference remains limited in a single case.

## Data Availability

The datasets presented in this article are not readily available because of ethical and privacy restrictions. Requests to access the datasets should be directed to the corresponding author.
